# The Ca^2+^ sensor proteins CML37 and CML42 antagonistically regulate plant stress responses by altering phytohormone signals

**DOI:** 10.1007/s11103-021-01184-2

**Published:** 2021-09-01

**Authors:** Monika Heyer, Sandra S. Scholz, Michael Reichelt, Grit Kunert, Ralf Oelmüller, Axel Mithöfer

**Affiliations:** 1 Research Group Plant Defense Physiology, Max Planck Institute for Chemical Ecology, Hans-Knöll-Straße 8, 07745 Jena, Germany; 2 Department for Plant Physiology, Matthias Schleiden Institute, Friedrich Schiller University, Dornburger Straße 159, 07743 Jena, Germany; 3 Department of Biochemistry, Max Planck Institute for Chemical Ecology, Hans-Knöll-Straße 8, 07745 Jena, Germany

**Keywords:** Jasmonates, Abscisic acid, Drought, Herbivory, Necrotrophic pathogenes, Defense, Calcium, Glucosinolates, *Spodoptera littoralis*, *Alternaria brassicicola*

## Abstract

**Key message:**

Calmodulin-like-proteins (CML) belong to a family of calcium-sensing proteins that are unique for plants and involved in many different developmental and stress-related reactions. In defense against herbivory, some pathogens and drought, CML37 acts as a positive and CML42 as a negative regulator, respectively. We provide evidence that both CMLs act antagonistically in the regulation of induced defense responses. A double knock-out line, *cml37* x *cml42*, thus shows wild-type phenotypes upon all kind of stresses we used.

**Abstract:**

A transient increase in the cytosolic calcium concentration is one of the first reactions that can be measured in plant cells upon abiotic as well as biotic stress treatments. These calcium signals are sensed by calcium binding proteins such as calmodulin-like proteins (CMLs), which transduce the sensed information into appropriate stress responses by interacting with downstream target proteins. In previous studies, CML37 has been shown to positively regulate the plants’ defense against both the insect herbivore *Spodoptera littoralis* and the response to drought stress. In contrast, CML42 is known to negatively regulate those two stress responses. Here, we provide evidence that these two CMLs act antagonistically in the regulation of induced responses directed against drought and herbivory stress as well as in the defense against the necrotrophic pathogen *Alternaria brassicicola*. Both CMLs shape the plant reactions by altering the phytohormone signaling. Consequently, the phytohormone-regulated production of defensive compounds like glucosinolates is also antagonistically mediated by both CMLs. The finding that CML37 and CML42 have antagonistic roles in diverse stress-related responses suggests that these calcium sensor proteins represent important tools for the plant to balance and fine-tune the signaling and downstream reactions upon environmental stress.

## Introduction

Plants are faced with a multiplicity of environmental changes at the same time. In order to adapt to their changing environment, each of these stimuli needs to be perceived and translated into an appropriate response via complex signaling networks. Calcium (Ca^2+^) as a second messenger plays a central role in these signaling networks. In response to various abiotic and biotic stimuli, changes in the cytosolic Ca^2+^ concentration are reported, e.g. after drought and salinity stress (Knight et al. 1997), extreme temperature fluctuations (Knight et al. 1991; Plieth et al. 1999), light (Shacklock et al. 1992), mechanical stimulation (Knight et al. 1991) as well as after interaction with symbionts (Ehrhardt et al. 1996; Vadassery et al. 2009), pathogens-derived elicitors (Knight et al. 1991) or herbivores (Maffei et al. 2004). Each of those stimuli induces a Ca^2+^ oscillation in the cell, the so called Ca^2+^ signature, that can differ in location, duration, amplitude and frequency, reflecting the strength and the type of the stimulus (McAinsh et al. 1997). Together with other cellular messengers, such as reactive oxygen species, pH changes and membrane potential changes, these complex spatio-temporal Ca^2+^ signatures form a code, transferring specific information about the environment into the plant cell (McAinsh et al. 1997; Plieth 2016). In order to translate this code into the respective response of the plant, the cellular changes need to be sensed.

Calcium (Ca^2+^) oscillations are sensed by Ca^2+^ binding proteins. These proteins possess Ca^2+^ binding motifs, consisting out of two helices, the E- and the F-helix, connected via a loop-structure and thus called EF-hand (Kretsinger and Nockolds 1973). An EF-hand binds a single Ca^2+^ ion (Kretsinger and Nockolds 1973), leading to a conformational change of the Ca^2+^ sensor protein that allows subsequently the interaction with a certain target. Some sensors, so-called sensor responders, have additionally enzymatic domains, like the Ca^2+^-dependent protein kinases (CDPKs) in plants that are activated upon binding of Ca^2+^. However, most Ca^2+^ sensing proteins just possess EF-hands as functional domains and thus are dependent on an interacting protein to transduce the sensed signal into a response. In plants three major groups of these sensor relays are distinguished: calmodulins (CaMs), calmodulin-like proteins (CMLs) and calcineurin B-like proteins (CBLs) (Sanders et al. 2002).

Amongst them, the family of CMLs is of special interest for decoding calcium signatures upon stress in plants, since they are unique to the plant kingdom (Bender and Snedden 2013). Further, in comparison to the related CaMs, CMLs display a much more distinctive expression pattern upon various stress treatments in *Arabidopsis thaliana*, suggesting that they might play a role in decoding calcium signals upon stress in plants (McCormack et al. 2005). For a few members of the CML family, there is also evidence that they mediate responses to various stresses in *A. thaliana*, although for most CMLs a functional characterization is still missing. Recently it was shown that CML41 reduces bacterial infection of *Pseudomonas syringae* by regulating the closure of plasmodesmata (Xu et al. 2017). Also CML8, CML9 and CML24 are known to positively regulate the immune response to *P. syringae* (Leba et al. 2012; Ma et al. 2008; Zhu et al. 2017). Besides, CML9 and CML24 have been shown to mediate the salt stress response in *A. thaliana* (Delk et al. 2005; Magnan et al. 2008) and thus seem to play a role in abiotic as well as biotic stress regulation. Similarly, CML37 and CML42 are known to be important in regulating both, abiotic and biotic stress responses. They mediate the defense against the lepidopteran herbivore *Spodoptera littoralis* and the drought stress reaction of *A. thaliana* (Scholz et al. 2014, 2015; Vadassery et al. 2012). In both stress treatments it has been shown that CML37 and CML42 act via altering the phytohormone signaling in the plant. In response to *S. littoralis* feeding, loss-of-function mutants of CML37 accumulated less jasmonates, leading to a higher susceptibility of the plant to the herbivore (Scholz et al. 2014). In contrast, knock out mutants of CML42 showed an upregulation of jasmonate-dependent defense responses, but a wild type-like jasmonate elevation, suggesting hypersensitivity to jasmonates and causing a higher resistance to the herbivore (Vadassery et al. 2012). Similarly, *cml42* accumulated higher levels of abscisic acid (ABA) upon drought conditions and thus was more resistant to drought, whereas *cml37* displayed no ABA response at all and was highly more susceptible (Scholz et al. 2015; Vadassery et al. 2012)*.*

Since CML42 turned out to be a negative regulator of both herbivore and drought stress tolerance and CML37 a positive one, it was hypothesized that they might be antagonists in regulating these stress responses (Scholz et al. 2014, 2015). To examine the interplay of CML42 and CML37, we constructed a double knock out mutant line and analyzed the response of this line upon drought and *S. littoralis* feeding in order to directly connect this investigation with the former studies. We show that effects of *cml37* abolish the effects of *cml42* in the double knock out mutant line and vice versa, verifying that CML37 and CML42 act antagonistically in regulating both stress responses. Further, we included infection with the necrotrophic fungal pathogen *A. brassicicola* in order to extend the study with a different type of stress. We demonstrate that CML37 and CML42 also regulate the defense against *A. brassicicola* antagonistically, suggesting a general antagonistic role of CML37 and CML42 in the regulation of the jasmonate-dependent defense responses. By studying a double knock out mutant of both CMLs we are now able to refine the roles of CML37 and CML42 in balancing different stress responses.

## Materials and methods

### Plant materials

*Arabidopsis* plants were grown under short day conditions at the MPI CE Jena in round pots with 10 cm diameter as described in Heyer et al. (2018). The double knock out mutant line *cml37* × *cml42* was obtained by crossing *cml37-1* (SALK_011488C) (Scholz et al. 2014) and *cml42* (SALK_041400C) (Dobney et al. 2009; Vadassery et al. 2012). Plants were selected by genotyping and selection was proven by RT-PCR. Experiments were performed with the F4 and F5 progeny of the crossed plants. *Arabidopsis thaliana* ecotype Col-0 was used as control.

Fungus treatments were performed at the FSU Jena and plants were grown on plates as described in Johnson et al. (2013). After 21 days plants were transferred to soil and further cultivated as described in Heyer et al. (2018).

### Insect rearing and oral secretion (OS) collection

*Spodoptera littoralis* eggs were obtained from Syngenta Crop Protection AG (Stein, Switzerland). Larvae were reared on an artificial diet based on ground beans modified after Bergomaz and Boppré (1986) at 23–25 °C and with 14 h photoperiod. Modifications of the diet composition are described in Heyer et al. (2018). For collection of OS, fourth instar larvae were starved overnight and were allowed to feed on the respective plant genotype for one day. OS was collected on ice and centrifuged at 13,000 rpm for 5 min after collection (Vadassery et al. 2012). It was stored at − 80 °C until use.

### Fungal growth

*Alternaria brassicicola* (FSU-218) was obtained from Jena Microbial Resource Center (Jena, Germany). Fungus was grown according to Heyer et al. (2018).

### Plant treatments

All herbivore-related experiments were done with 5–6 week old plants. One week feeding assays were performed with first instar larvae as described in Vadassery et al. (2012) (see insect biomass assay). For short term feeding assays fourth instar larvae were used. To ensure sufficient feeding, larvae were starved 12 h prior the assays and three larvae were allowed to feed on one plant (Scholz et al. 2014; Vadassery et al. 2012). OS treatments were done as described in Vadassery et al. (2012).

Four week old plants were used for drought stress assays. Drought was applied for 1 or 2 weeks. If applied for 2 weeks, plants were re-watered after first week of drought stress as described in Scholz et al. (2015). Control and mutant plants were kept randomly distributed on the same tray to minimize experimental variation.

Plant material collected for metabolite quantification or RT-PCR was immediately frozen in liquid nitrogen and stored at − 80 °C until extraction.

For pathogen treatments detached, fully expanded leaves of 5–6 week old plants were used. *Alternaria* treatments were carried out as described in Heyer et al. (2018).

### Genotyping

Single leaves of 3 week old plants were cut and immediately frozen in liquid nitrogen. Plant material was ground using 2010 Geno/Grinder® (SPEX®SamplePrep, Metuchen USA). To avoid defrosting of the samples, they were stored in precooled aluminum racks throughout grinding process. DNA was extracted after a modified protocol of Konieczny and Ausubel (1993). Modifications are described in Heyer et al. (2018). PCR was performed using native Taq DNA polymerase and 10 mM dNTP Mix from Invitrogen™ by Thermo Fisher Scientific (Carlsbad, USA). PCR mix was prepared according to manufacturers’ protocol. The total reaction volume was scaled down to 10 µl, including 1.5 µl of template. Primers published in Scholz et al. (2014) and Vadassery et al. (2012) were used.

### Semi-quantitative reverse transcription (RT)-PCR

Treated leaves were sampled and ground as described above. RNA was isolated using TRIzol® Reagent (Invitrogen™ by Thermo Fisher Scientific, Carlsbad, USA). Extraction was performed according to the manufacturers’ protocol with modifications as described in Heyer et al. (2018). To avoid DNA contamination, extracted RNA was treated with TURBO DNase (TURBO DNA-*free*™ Kit, Invitrogen™ by Thermo Fisher Scientific, Vilnius, Lithuania). PCR was done as described above. *ACTIN2* was used as housekeeping gene. Primers for *CML37* as published in Scholz et al. (2014) and *CML42* and *ACTIN2* as described in Vadassery et al. (2012) were used.

### Phytohormone quantification

Phytohormones were extracted from treated leaves using the protocol described in Jimenez-Aleman et al. (2015) with slight modifications. Approximately 250 mg of ground leaf material was extracted using 1.5 ml methanol containing 60 ng D_6_-ABA (Santa Cruz Biotechnology, Santa Cruz, USA), 60 ng of D_6_-jasmonic acid (HPC Standards GmbH, Cunnersdorf, Germany), 60 ng D_4_-salicylic acid (Sigma-Aldrich, St. Louis, USA) and 12 ng of jasmonoyl-^13^C_6_-isoleucine [synthesized as described in Kramell et al. (1988)] as internal standard. Phytohormone analysis was performed according to the protocol of Vadassery et al. (2012). Protocol was modified as follows. For herbivore treated samples chromatography was performed on an Agilent 1260 HPLC system (Agilent Technologies, Santa Clara, USA), for drought stress samples chromatographic separation was done on an Agilent 1200 HPLC (Agilent Technologies, Santa Clara, USA) using a Zorbax Eclipse XDB-C18 column (50 × 4.6 mm, 1.8 µm, Agilent Technologies, Santa Clara, USA) in both cases. Water containing 0.05% formic acid and acetonitrile were employed as mobile phases A and B, respectively. The elution profile for herbivore treated samples was: 0–0.5 min, 5% B; 0.5–9.5 min, 5–42% B; 9.5–9.51 min, 42–100% B; 9.51–12 min 100% B and 12.1–15 min, 5% B. In case of drought stress treated samples elution profile was: 0–0.5 min, 10% B; 0.5–4.0 min, 10–90% B; 4.0–4.02 min, 90–100% B; 4.02–4.5 min, 100% B and 4.51–7.0, min 10% B. Flow rate was kept at 1.1 ml/min and column temperature was maintained at 25 °C. Mass spectrometry of herbivore treated samples was performed on an API 5000 tandem mass spectrometer (Applied Biosystems™, Darmstadt, Germany) and on an API 3200 tandem mass spectrometer (Applied Biosystems™, Darmstadt, Germany) in case of drought stressed samples. Both spectrometers were equipped with a Turbo spray ion source operated in negative ionization mode. The ion spray voltage was maintained at − 4500 eV. The turbo gas temperature was set at 700 °C. Nebulizing gas was set at 60 psi, curtain gas at 25 psi, heating gas at 60 psi, and collision gas at 7 psi. The following analyte parent ion → product ion fragmentations were used for multiple reaction monitoring (MRM): mass-to-charge ratio (*m*/*z*) 263.0 → 153.2 [collision energy (CE) − 22 V; declustering potential (DP) − 35 V] for ABA; *m*/*z* 269.0 → 159.2 (CE − 22 V; DP − 35 V) for D_6_-ABA; *m*/*z* 209.1 → 59.0 (CE − 24 V; DP − 35 V) for jasmonic acid (JA); *m/z* 215.1 → 59.0 (CE − 24 V; DP − 35 V) for D_6_-JA; *m*/*z* 136.9 → 93.0 (CE − 22 V; DP − 35 V) for salicylic acid (SA); *m*/*z* 140.9 → 97.0 (CE − 22 V; DP − 35 V) for D_4_-SA; *m*/*z* 290.9 → 165.1 (CE − 24 V; DP − 45 V) for *cis*-12-oxophytodienoic acid (OPDA), *m*/*z* 322.2 → 130.1 (CE − 30 V; DP − 50 V) for jasmonoyl isoleucine (JA-Ile); *m*/*z* 328.2 → 136.1 (CE − 30 V; DP − 50 V) for JA-^13^C_6_-Ile. Both Q1 and Q3 quadrupoles were maintained at unit resolution. Analyst 1.5 software (Applied Biosystems™, Darmstadt Germany) was used for data acquisition and processing. Linearity in ionization efficiencies was verified by analyzing dilution series of standard mixtures. Phytohormones were quantified relative to the signal of their corresponding internal standard. For quantification of OPDA the internal D_6_-JA standard was used applying experimental-determined response factors of 0.5 respectively. The response factor was determined by analyzing a mixture of *cis*-OPDA [kindly provided by W. Boland, MPI for Chemical Ecology, Jena, Germany; synthesized as described in Shabab et al. (2014)] and D_6_-JA all at the same concentration. For JA-Ile quantification after herbivory only the peak of the endogenous bioactive ( +)-7-*iso*-jasmonoyl-L-isoleucine (Fonseca et al. 2009) was used.

### Quantification of glucosinolates

Whole *Arabidopsis* rosettes where collected and freeze dried, to avoid fast degradation of glucosinolates. Freeze-dried samples were ground to a fine powder in uncooled racks in 2010 Geno/Grinder® (SPEX®SamplePrep, Metuchen USA). Extraction was performed according to Burow et al. (2006) with some modifications. For each sample, approximately 25 mg per sample were extracted in 1 ml 80% methanol containing 50 µM 4-hydroxybenzylglucosinolate [isolated from *Sinapis alba* seeds according to Thies (1988)] as internal standard. Samples were mixed for 10 min at room temperature and pelleted by centrifugation. 800 µl of the supernatant was transferred to columns containing 28 mg DEAE Sephadex A25 (Sigma-Aldrich, St. Louis, USA) each. Columns were prewashed with 800 µl water and 500 µl 80% methanol. After loading the samples, columns were washed with 500 µl 80% methanol and twice with 1 ml water. Afterwards they were rinsed with 500 µl 0.02 M MES buffer (pH 5.2) and 30 µl of sulfatase (from *Helix pomatia*, Sigma Aldrich, St. Louis, USA) was applied to the columns. Sulfatase was prepared according to Graser et al. (2000). Columns were incubated for desulfation at room temperature overnight and desulfoglucosinolates were eluted with 500 µl water. Desulfoglucosinolates were analyzed using HPLC/UV and quantified as described in Vadassery et al. (2012).

### Chlorophyll fluorescence measurements

Chlorophyll fluorescence parameters was measured in a FluorCam FC 800-C (Psi, Drasov, Czech Republic) as described in Heyer et al. (2018).

### Statistical analysis

Statistics were performed using R 3.5.1 (R Development Core Team 2018) and SigmaPlot 11.0 (Systat Software 2008). Differences in larval weight of *S. littoralis* were tested by Wilcoxon-test. Differences in phytohormone concentration between two genotypes were determined with Wilcoxon-test or t-test depending on the homogeneity and normal distribution of the data. In order to test whether the glucosinolate content, chlorophyll fluorescence and ABA content differed between different genotypes with different treatments, two-way ANOVAs was applied, followed by Tukey tests as post-hoc test if necessary In case of variance inhomogeneity, generalized least square method [gls from the nlme library (Pinheiro et al. 2018)] with varIdent function was applied instead of a two-way ANOVA. Whether the different variance of genotype, treatment, or the combination of both factors should be incorporated into the model was determined by comparing different variance structure models, selecting for the model with the lowest Akaike Information Criteria (AIC) (Zuur et al. 2009). The influence of the different genotypes, treatments and the interaction of both was determined by likelihood ratio tests following the backward selection protocol of Zuur et al. (2009). To test for differences among the groups factor level reduction was used (Crawley 2013). In order to obtain normal distribution of residuals, data were transformed before applying the statistic test, when it was necessary. The respective statistical test and transformation method is mentioned in the respective tables. Total number of replicates (n) is indicated in the figure legend. All experiments shown are repeated at least two times independently.

## Results

### Construction of the *cml37* × *cml42* double knock out line

**Fig. 1:**
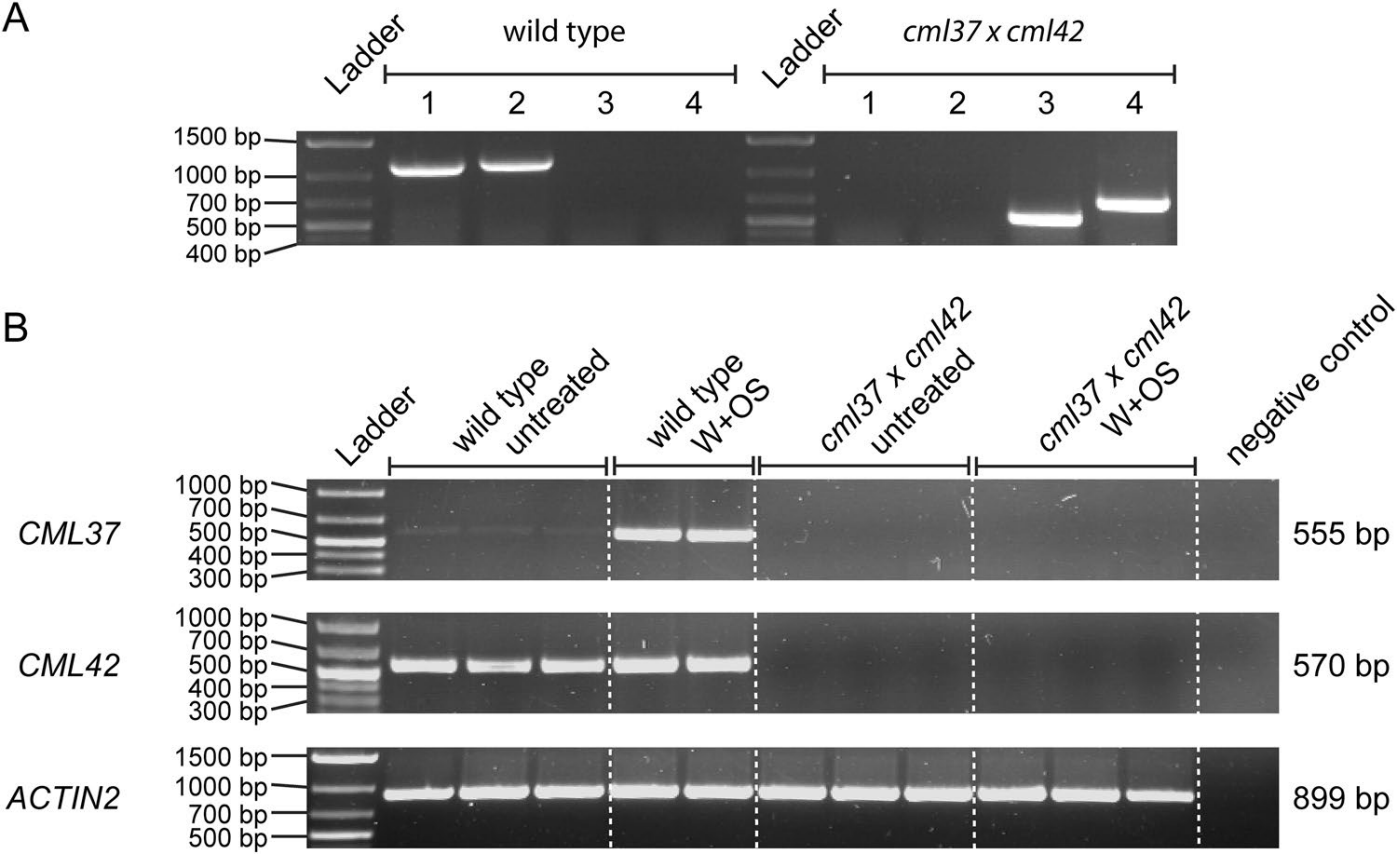
Genetic characterization of *cml37* × *cml42*. **A** Results of Genotyping *cml37* × *cml42*. Different numbers indicate different Primer-sets used: (1) *CML42* RP + *CML42* LP (expected product size: 1120 bp) to verify the presence of the wild type *CML42* (2) *CML37* RP + *CML37* LP (expected product size: 1158 bp) to verify the presence of the wild type *CML37* (3) *CML42* RP + LBb1.3 (expected product size: 456–756 bp) to verify the presence of the T-DNA insertion in *CML42* (4) *CML37* RP + LBb1.3 (expected product size: 599–899 bp) to verify the presence of the T-DNA insertion in *CML37* (B) RT-PCR to confirm the absence of the *CML37* and *CML42* transcripts in *cml37* × *cml42*. Plants were wounded with a pattern wheel and treated with oral secretion (W + OS) for 1 h or were used untreated. Water was used as negative control. Expected product size is indicated to the right of the gel pictures. *ACTIN2* expression was used as quantitative control

To investigate the possible antagonism of CML37 and CML42, a loss-of-function mutant of both proteins was generated by crossing the T-DNA insertion lines *cml37-1* (Scholz et al. 2014) and *cml42* (Dobney et al. 2009; Vadassery et al. 2012). The homozygosity of the crossed line was confirmed by genotyping (Fig. 1A). For both genes the product for the intact gene was not detectable, whereas the product including the left border of the T-DNA was detectable in both cases (Fig. 1A), showing that *cml37* × *cml42* is homozygous.

Further, the absence of *CML37* and *CML42* transcript was confirmed by RT-PCR. Since the constitutive expression level of *CML37* in adult leaves is comparatively low (McCormack et al. 2005), plants were wounded and treated with *S. littoralis* OS to stimulate the expression of the CMLs. Whereas both CMLs are expressed in untreated and treated wild type plants, no product was detected in case of the double knock out mutant (Fig. 1B), confirming that it is a loss of function of both CMLs.

### *S. littoralis* performance is not affected in *cml37* × *cml42*

**Fig. 2:**
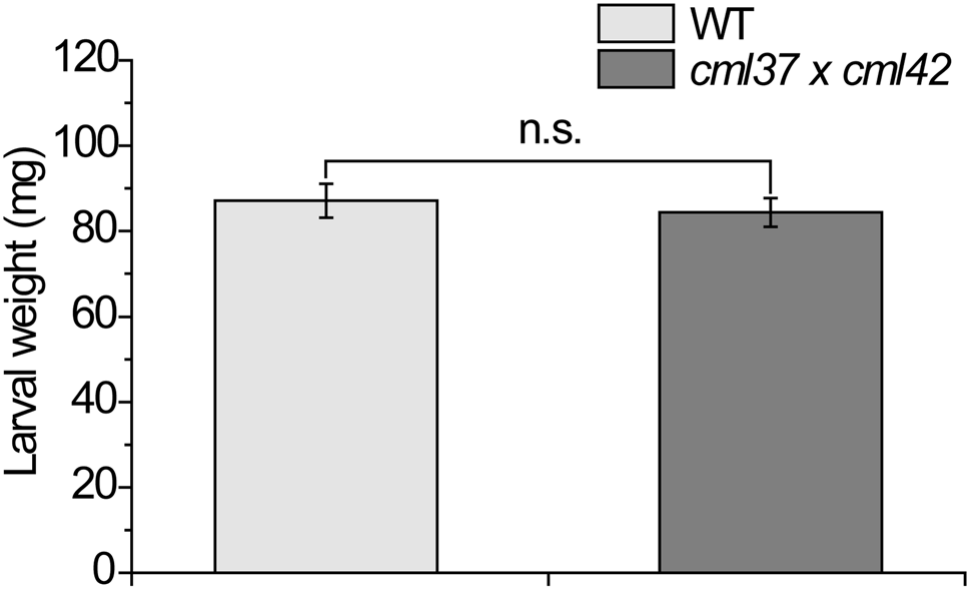
Feeding performance of *S. littoralis* larvae on *cml37* × *cml42*. Larval weight gain in mg ± Standard error (SE) after 1 week of feeding on either *cml37* × *cml42* or wild type (WT) plants. First instar *S. littoralis* larvae were pre-weighed to reduce experimental variation. Three larvae were placed on each plant. After 1 week of feeding, larval weight was determined. Experiment was repeated five times independently [n = 138 (Larvae on WT), n = 140 (Larvae on *cml37* × *cml42*)]. Larval weights of both genotypes were compared; n.s. means not significant (p = 0.9589)

To test the hypothesis of the possible antagonism of CML37 and CML42 in the herbivore defense, the *cml37* × *cml42* mutant line was used for insect performance assays. First instar *S. littoralis* larvae were allowed to feed on wild type and mutant plants for 1 week. The insect performance was evaluated by the gain of weight. Results are presented in Fig. 2. After 1 week, *S. littoralis* larvae gained as much weight on *cml37* × *cml42* as on wild type plants, suggesting the positive effect of *cml37* and the negative effect of *cml42* on the larval weight gain (Scholz et al. 2014; Vadassery et al. 2012) compensate each other in the double knock out mutant. Thus, both CMLs seem to be antagonistic in regulating the herbivore defense of *A. thaliana*.

### *cml37* × *cml42* displays a wild type-like phytohormone response after herbivory

**Fig. 3:**
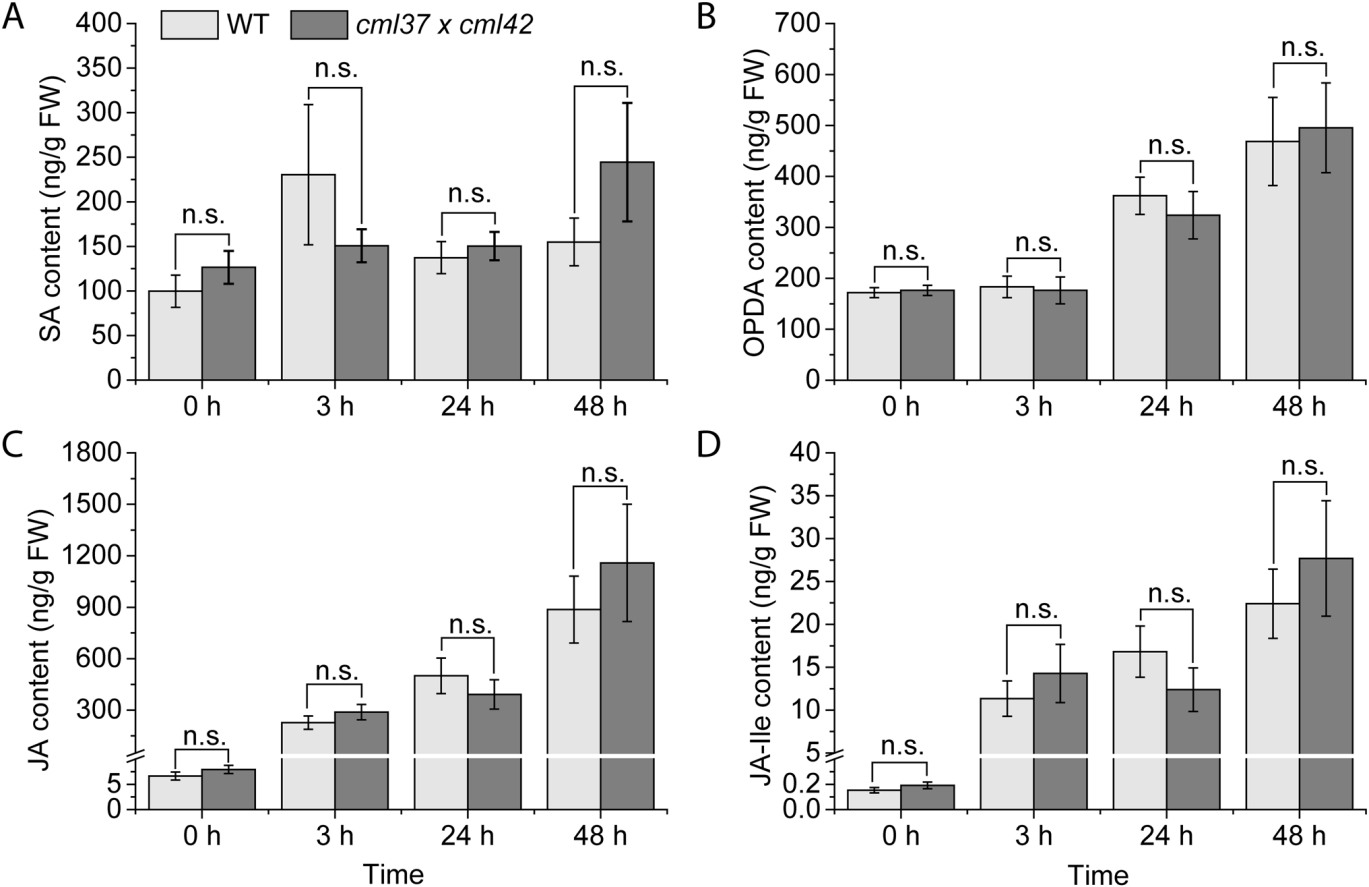
Phytohormone contents of *cml37* × *cml42* after feeding of *S. littoralis* larvae. Contents of **A** SA, **B** OPDA, **C** JA, and **D** JA-Ile in ng/g fresh weight (FW) ± SE in *cml37 x cml42* and WT plants after *S. littoralis* feeding. Larvae were allowed to feed for 3 h, 24 h and 48 h on the plants. Phytohormones were analyzed from local fed leaves. Untreated plants were used as controls (0 h). Experiment was repeated four times independently (n ≥ 10). Phytohormone contents of both genotypes within the time point were compared; n.s. means not significant. The respective p-values are given in Table 1. Legend for color code see (**A**)

In former studies, the higher susceptibility of *cml37* to *S. littoralis* was shown to be caused by a lower level of the jasmonate-precursor OPDA and the active jasmonate JA-Ile (Scholz et al. 2014). However, in *cml42*, there was no difference in phytohormone concentration between the wild type and the mutant (Vadassery et al. 2012). Thus, we investigated the phytohormone levels after *S. littoralis* feeding on *cml37* × *cml42.* Similar to both single mutant lines, the levels of SA and JA did not change in the double knock out mutant line compared to the wild type (Fig. 3A, C, Table 1). However, in contrast to the single mutant lines, *cml37* × *cml42* plants also displayed wild type-like OPDA and JA-Ile concentrations in non-treated controls and after *S. littoralis* feeding (Fig. 3B, D, Table 1). Even though CML42 was shown to have no effect on phytohormone levels itself (Vadassery et al. 2012), *cml42* is able to rescue the negative effect of *cml37* on the OPDA and JA-Ile levels in *cml37* × *cml42* after insect feeding.

**Table 1:** Results of Wilcoxon and unpaired t-tests for analyzing differences in phytohormone content after herbivory

Phytohormone	Time point (h)	Test	p-value
SA	0	t-test	0.307
	3	Wilcoxon	0.3245
	24	t-test	0.592
	48	Wilcoxon	0.5116
OPDA	0	t-test	0.7577
	3	Wilcoxon	0.5202
	24	t-test	0.5208
	48	Wilcoxon	0.9725
JA	0	Wilcoxon	0.1434
	3	Wilcoxon	0.4124
	24	t-test	0.4296
	48	Wilcoxon	0.9725
JA-Ile	0	Wilcoxon	0.5742
	3	Wilcoxon	0.6782
	24	t-test	0.271
	48	t-test	0.5196

### Induction of secondary metabolites is altered in *cml37* × *cml42*

**Fig. 4:**
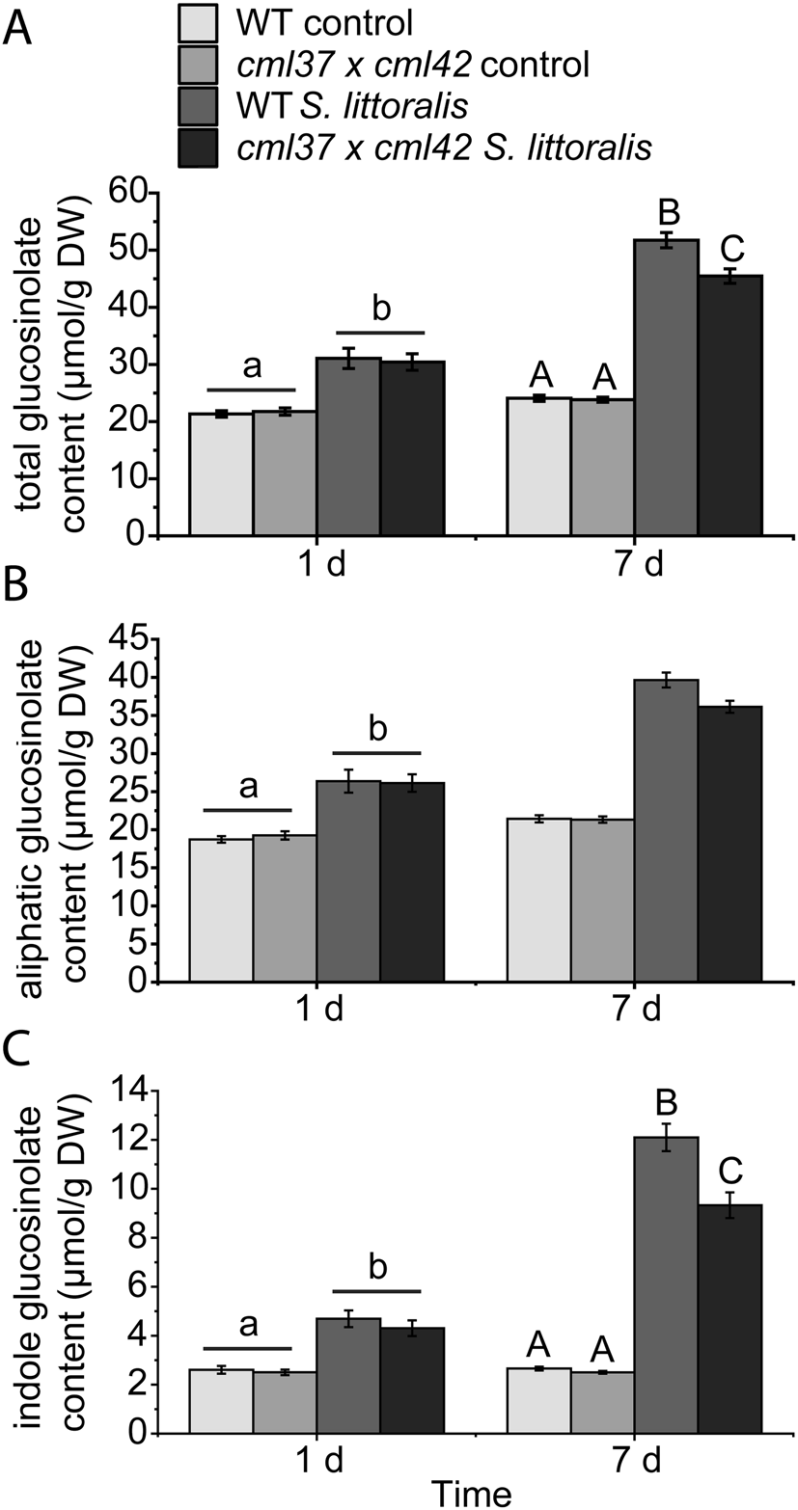
Glucosinolate content of *cml37* × *cml42* after feeding of *S. littoralis* larvae. **A** Total glucosinolate, **B** aliphatic glucosinolate and **C** indole glucosinolate contents of WT and *cml37* × *cml42* plants in µmol/g dry weight (DW) ± SE after 1 days (5 week old plants) and 7 days (6 week old plants) of *S. littoralis* feeding. Untreated plants were used as controls. Glucosinolates were extracted from whole *Arabidopsis* rosettes. Experiment was repeated at least four times independently (n ≥ 14). Differences between glucosinolate contents of controls, treated samples and genotypes were determined within one time point by two-way ANOVA (**A**, **B**) and generalized least square method (**C**). Differences between the groups are indicated by different letters above the bars (p-value ≤ 0.05). Details about all the statistic tests including the respective statistical values and especially for 7 days aliphatic glucosinolates, are listed in Table 2. Legend for color code see (**A**)

Vadassery et al. (2012) published that the higher resistance of *cml42* to insect feeding is, *inter alia*, due to a higher constitutive level of defensive compounds, the glucosinolates. On the other hand, loss-of-function mutants of CML37 displayed constitutive and induced glucosinolate levels that were comparable to those of wild type plants (Scholz et al. 2014). In order to investigate if *cml37* is able to rescue the glucosinolate phenotype of *cml42*, we measured the glucosinolate content of *cml37* × *cml42* before and after *S. littoralis* feeding. There were no differences in the total glucosinolate levels of untreated 5–6 week-old *cml37* × *cml42* and wild type plants (Fig. 4A, Table 2). Since the higher total constitutive glucosinolate content in *cml42* was due to an increased level of aliphatic glucosinolates (Vadassery et al. 2012), we further distinguished into aliphatic and indole glucosinolates. Untreated 5 and 6 week-old *cml37* × *cml42* plants displayed a wild type-like constitutive level of aliphatic and indole glucosinolates, showing that *cml37* counteracts the effect on glucosinolates of *cml42* in the double knock out mutant line (Fig. 4B, C, Table 2). Further, no differences in the induced total, aliphatic or indole glucosinolate content were measured after 1 day feeding between *cml37* × *cml42* and wild type plants (Fig. 4, Table 2). However, after 1 week of feeding, the induction of glucosinolates in *cml37* × *cml42* was significantly lower than in wild type plants (Fig. 4A, Table 2). This seems to be due to both the lower level in aliphatic as well as in indole glucosinolates (Fig. 4B, C). Although the interaction of genotype and treatment was not significant in case of the aliphatic glucosinolates there is a clear tendency (Table 2). This result seems to be a secondary effect only detected in the double knock out mutant, since neither *cml37* nor *cml42* were impaired in the induction of glucosinolates (Scholz et al. 2014; Vadassery et al. 2012). However, the lower induction of glucosinolates after 7d does not cause better larval performance after 1 week (Fig. 2). Thus, it is unclear if this late reduction of the glucosinolates content in *cml37* × *cml42* is of functional relevance for the insect defense.

**Table 2:** Statistical values for the analysis of glucosinolates at different time points according to herbivory, plant genotype, and the interaction between herbivory and plant genotype

Glucosinolate at time point	Test	Transfor-mation/variance structure	Factor	p-value	F-/L-ratio	Post-hoc/factor level reduction (p ≤ 0.05)
Total glucosinolates 1 day	Two-way ANOVA	log_10_ x	T	< 0.001	80.687	Control < feeding
			G	0.967	0.002	Not tested
			T×G	0.660	0.196	Not tested
Total glucosinolates 7 days	Two-way ANOVA	log_10_ x	T	< 0.001	826.717	Control < feeding
			G	0.005	8.366	*cml37* × *cml42* < WT
			T×G	0.016	6.150	WT control = *cml37* × *cml42* control < *cml37* × *cml42* feeding < WT feeding
Aliphatic glucosinolates 1 day	Two-way ANOVA	1/√x	T	< 0.001	71.162	Control < feeding
			G	0.695	0.155	Not tested
			T×G	0.738	0.113	Not tested
Aliphatic glucosinolates 7 days	Two-way ANOVA	log_10_ x	T	< 0.001	653.754	Control < feeding
			G	0.033	4.740	*cml37* × *cml42* < WT
			T×G	0.053	3.874	Not tested
Indole glucosinolates 1 day	Generalized least squares	*Different variances amongst T*	T	< 0.0001	*35.281*	Control < feeding
			G	0.416	*0.661*	Not tested
			T×G	0.561	*0.338*	Not tested
Indole glucosinolates 7 days	Generalized least squares	*Different variances amongst T*	T	< 0.0001	*85.695*	Control < feeding
			G	0.046	*3.996*	*cml37* × *cml 42* < WT
			T×G	0.001	*10.478*	WT control = *cml37* × *cml42* control < *cml37* × *cml42* feeding < WT feeding

Either two-way ANOVA or generalized least square method was used to determine difference within one time point. In case of two-way ANOVA Tukey test was used as post-hoc test. Depending which statistical test was used F-values or Likelihood ratios (L-ratio) are given. L-ratios are given in italics. To account for the variance heterogeneity of the residuals data were either transformed before a two-way ANOVA or generalized linear models with the varIdent variance structure were used. Variance structures are given in italics

*T* treatment, *G* genotype,* T*×*G* interaction treatment and genotype

### *cml37* and *cml42* display a different susceptibility to *A. brassicicola*

**Fig. 5:**
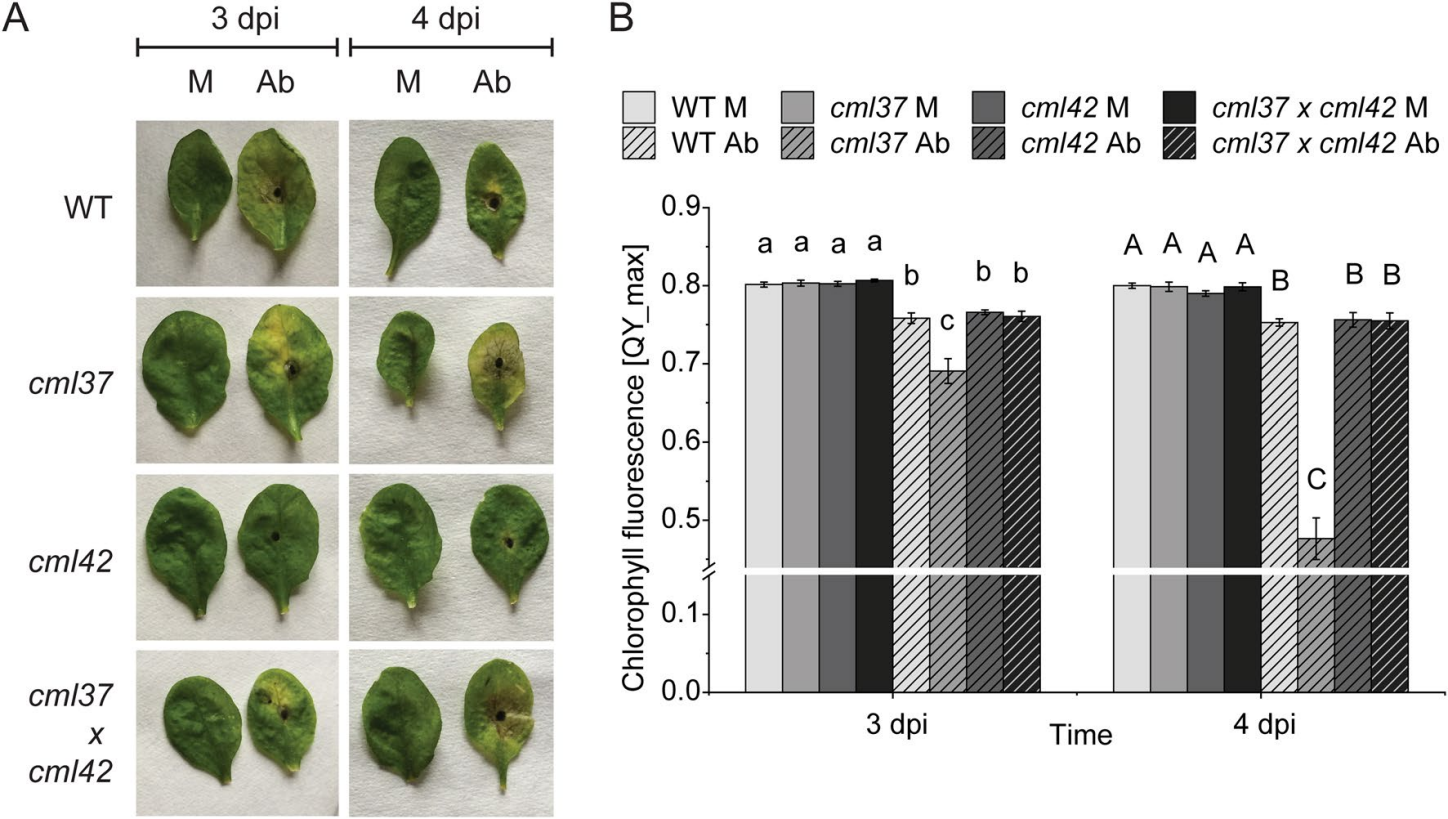
Different susceptibility of *cml37*, *cml42* and *cml37* × *cml42* to *A. brassicicola*. **A** Necrotic lesion phenotype and **B** chlorophyll fluorescence (QY_max) ± SE of *cml37*, *cml42*, *cml37* × *cml42* and WT 3 and 4 day post-inoculation (dpi) with *A. brassicicola* spore suspension (Ab) or 0.01% Tween as mock (M). Plants shown are representative. Experiment was repeated at least two times independently [n ≥ 12 (3 dpi), n ≥ 6 (4 dpi)]. Differences in the chlorophyll fluorescence were tested by generalized least square method within the time point. Differences between the groups are indicated by different letters above the bars (p-value ≤ 0.05). Detailed information about the statistic tests and statistical values are listed in Table 3

Besides mediating the defense against herbivores, jasmonates are also important signaling components in the defense against necrotrophic pathogens (Glazebrook 2005; Thomma et al. 1998). Since both CML37 and CML42 are regulating the defense against the herbivore *S. littoralis* by affecting the jasmonate pathway (Scholz et al. 2014; Vadassery et al. 2012), we examined whether they are influencing the resistance to the necrotrophic fungus *A. brassicicola* as well. In line with the results from the herbivore assays, both *cml* mutants displayed an antagonistic phenotype. Whereas *cml37* was much more susceptible to the fungus treatment, *cml42* seemed to be more resistant than the wild type (Fig. 5A). After 3 days of treatment, necrotic lesions were slightly larger on *cml37* than on wild type leaves (Fig. 5A). After 4 days, the necrotic area was even covering nearly the whole *cml37* leaf, whereas just a part of the wild type leaf was necrotic (Fig. 5A). On the other hand, lesions on *cml42* leaves were smaller than on wild type plants at both time points (Fig. 5A). Interestingly *cml37* × *cml42* displayed an intermediate lesion forming phenotype that was comparable to wild type plants (Fig. 5A), suggesting again that the negative effect of *cml37* and the positive effect of *cml42* are neutralizing each other in the double mutant.

In addition, the chlorophyll fluorescence of the leaves was measured to determine the differences in susceptibility. Corresponding to the phenotype, *cml37* leaves showed lower chlorophyll fluorescence than wild type plants after fungi treatment (Fig. 5B, Table 3). Surprisingly, the chlorophyll fluorescence of *cml42* was similar to those of wild type leaves (Fig. 5B, Table 3), although the necrotic area seemed to be smaller on mutant leaves (Fig. 5A). However, in *cml37* × *cml42* the chlorophyll fluorescence was comparable to wild type plants as well (Fig. 5B, Table 3), implying that *cml42* can recover the strong negative impact of *cml37*. Thus, CML37 and CML42 seem to regulate the defense against *A. brassicicola* antagonistically as well.

**Table 3:** Results of the generalized least square for analyzing the differences in the chlorophyll fluorescence

Time point	Factor	p-value	L-ratio
3 dpi	T	< 0.001	85.305
	G	0.507	2.330
	T×G	0.002	14.440
4 dpi	T	< 0.001	39.834
	G	0.406	2.911
	T×G	< 0.001	27.571

To define the variance structure, varIdent function was used, allowing a different variance of all groups

*T* treatment, *G* genotype, *TxG* interaction treatment and genotype

### Drought stress response is not altered in *cml37* × *cml42*

**Fig. 6:**
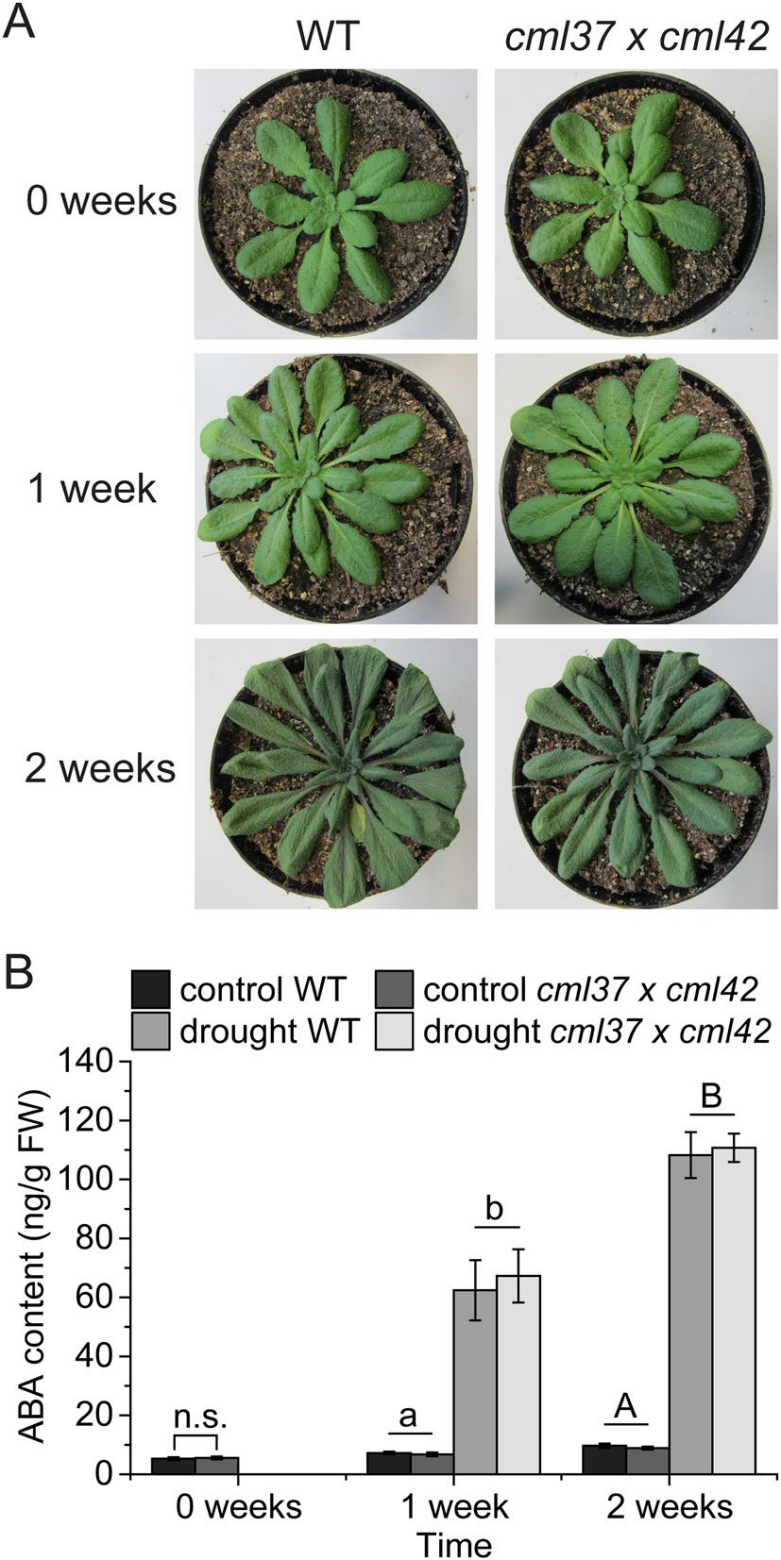
Drought stress responses of *cml37* × *cml42*. **A** Phenotypes and **B** ABA contents ± SE of *cml37* × *cml42* and WT plants after 1 or 2 weeks of drought stress. Plants exposed to drought for 2 weeks were re-watered once after 1 week. Untreated plants were used as controls. Plants shown are representative. Experiment was repeated four times independently (n ≥ 18). ABA levels of the genotypes at 0 weeks were compared by two-sample Wilcoxon test. The influence of genotype and drought stress was tested with a two-way ANOVA (1 week) or a generalized least square method (2 weeks). Differences between the groups are indicated by the letters above the bars (p-value ≤ 0.05). For p-values see Table 4; n.s. means not significant

An addition to their known function in jasmonate-mediated stress responses, CML37 and CML42 have been shown to regulate the drought stress response in *A. thaliana* (Scholz et al. 2015; Vadassery et al. 2012). It was observed that *cml37* plants are more sensitive to drought stress than wild type plants, while *cml42* seemed to be less affected (Scholz et al. 2015). The drought stress phenotype of both mutants was reflected in altered ABA levels: *cml42* displayed higher ABA levels after drought stress, whereas in *cml37* ABA was not induced at all upon drought (Scholz et al. 2015; Vadassery et al. 2012). In order to test if CML37 and CML42 are also antagonistically regulating the drought stress response, we investigated the response of *cml37* × *cml42* to drought. After 1 week without water, there was no difference in the drought stress phenotype between *cml37* × *cml42* and wild type plants (Fig. 6A). Plants were then watered and kept for a second week without water. Even after the second drought period, *cml37* × *cml42* was as tolerant as the wild type plants (Fig. 6A). Corresponding to that, *cml37* × *cml42* displayed wild type-like ABA levels among the whole treatment (Fig. 6B, Table 4). Again, the diverging effects of the single knock out mutants are balanced in the double knock out mutant line, suggesting that CML37 and CML42 are antagonists in the regulation of the drought stress response.

**Table 4:** Results of the statistic tests for analyzing the differences in the ABA content

Time point	Test	Transformation/variance structure	Factor	p-value	F-/L-ratio
0 weeks	Wilcoxon	–	–	0.5203	–
1 week	Two-way ANOVA	log_10_ x	T	< 0.001	1653.535
			G	0.235	1.438
			T×G	0.627	0.238
2 weeks	Generalized least square	*Different variances amongst all groups*	T	< 0.0001	*104.059*
			G	0.3809	*0.768*
			T×G	0.7126	*0.136*

Either Wilcoxon-test, two-way ANOVA or generalized least square method was used to determine difference within one time point. Depending which statistical test was used F-values or Likelihood ratios (L-ratio) are given. L-ratios are given in italics. To account for the variance heterogeneity of the residuals data were either transformed before a two-way ANOVA or generalized linear models with the varIdent variance structure were used. Variance structures are given in italics

*T* treatment, *G* genotype,* T*×*G* interaction treatment and genotype

## Discussion

The CMLs, a group of calcium sensors, has been shown to be responsible for sensing stress-mediated Ca^2+^ signals and regulating downstream defense reactions of the plant (Delk et al. 2005; Leba et al. 2012; Ma et al. 2008; Magnan et al. 2008; Scholz et al. 2014, 2015; Vadassery et al. 2012; Xu et al.^2017^; Zhu et al. 2017). However, not much is known about the interplay between the different CMLs in mediating different stress responses. Here we report about the antagonism of CML37 and CML42 in regulating the defense against herbivores and pathogens, as well as the drought stress response of *A. thaliana.*

### CML37 and CML42 regulate the defense to *S. littoralis* antagonistically

In previous studies CML37 was described as a positive regulator of defense against the insect herbivore *S. littoralis* whereas CML42 was shown to negatively influence this defense response (Scholz et al. 2014; Vadassery et al. 2012). Here we showed that if both CMLs are knocked out, the positive effect of CML37 and the negative effect of CML42 neutralize each other, suggesting that both CMLs act antagonistically. In feeding assays, *S. littoralis* larvae gained as much weight on the double knock out mutant line as on wild type plants (Fig. 2). In line with this result, the double knock out mutant displayed a wild type-like phytohormone response upon *S. littoralis* feeding (Fig. 3). However in former studies with the corresponding single knock out mutant lines, only *cml37* but not *cml42* had an effect on the level of phytohormones. In detail, *cml37* elevated less OPDA and JA-Ile upon insect feeding than the wild type, whereas *cml42* mutants accumulated the same amounts of jasmonates like the wild type (Scholz et al. 2014; Vadassery et al. 2012). Nevertheless, *cml42* is able to rescue the effect of *cml37* in the double knock out mutant (Fig. 3). This result suggests that *cml42* is able to positively influence the jasmonate accumulation. This positive influence of *cml42* might not have been displayed in the single mutant, since enhanced jasmonate levels are usually very costly to the plants and negatively affect their fitness. Plants that exhibit higher levels of jasmonates are often smaller and produce far less seeds (Baldwin 1998; Cipollini 2007). Thus, the plant might control the level of jasmonates and limit them via other regulators to a certain level. Nevertheless, it might be possible that there are secondary effects that just appear in the double knock out mutant, but not in the single mutants that explain the different effects of knocking out *CML42* in single and double knock out mutant on the jasmonate elevation.

A similar rescue effect of one of the single mutants in the double knock out line could be shown for the production of secondary metabolites. Here we investigated the levels of glucosinolates, a class of secondary metabolites produced especially as defensive compounds against herbivores in the Brassicaceae family (Halkier and Gershenzon 2006). It was known that *cml42* produced higher constitutive levels of mainly aliphatic glucosinolates (Vadassery et al. 2012). In *cml37* × *cml42* the constitutive levels of both aliphatic and indole glucosinolates were comparable to those of wild type plants (Fig. 4B, C), suggesting that *cml37* is able to counteract the effect of *cml42* on the constitutive levels of glucosinolates in *cml37* × *cml42*, although the indole glucosinolates were found to be slightly but significantly reduced after 7d of *S. littoralis* feeding (Fig. 4C). Thus, *cml37* might negatively influence the production of glucosinolates. However, such a negative effect on glucosinolate production was not measured in the single knock out line of *CML37*. It displayed a wild type-like glucosinolate pattern (Scholz et al. 2014). On the other side it is known that *cml37* alone negatively affected the biosynthesis of jasmonates (Scholz et al. 2014) and that the biosynthesis of glucosinolates is mainly regulated by the jasmonate pathway (Mewis et al. 2006; Schweizer et al. 2013). Thus *cml37* might also negatively influence the glucosinolate production. However, even *coi-1* mutants that are insensitive to jasmonates still possess a certain level of glucosinolates (Mewis et al. 2006; Schweizer et al. 2013). Clearly *cml37* is impaired in the jasmonate biosynthesis, but it displays a far less severe phenotype than *coi-1* (Scholz et al. 2014). Thus it is conceivable that *cml37* has a negative impact on the production of glucosinolates that is not pronounced in the single mutant, but strong enough to rescue *cml42* in the double knock out plants. However, also here secondary effects that occur in the double knock out mutant line cannot be fully excluded as a possible explanation of the rescue effect.

Since *cml37* is able to rescue effects of *cml42* and vice versa, we could clearly show that both CMLs regulate the defense against the insect herbivore *S. littoralis* antagonistically.

### The differential regulation of defense against necrotrophs by CML37 and CML42

Besides their role in the herbivore defense, some CMLs have been shown to be involved in the defense against pathogens (Leba et al. 2012; Ma et al. 2008; Xu et al. 2017; Zhu et al. 2017). However, most of the research is focusing on the biotrophic bacterial pathogen *P. syringae*, but less is known about the role of CMLs in the defense against necrotrophic pathogens. In gene expression studies *CML37* and *CML42* were both upregulated after infection with the necrotrophic fungus *Botrytis cinerea* (McCormack et al. 2005). Here, we showed that CML37 and CML42 are also important for the regulation of the defense against the necrotrophic fungus *A. brassicicola*. Whereas *cml37* was more susceptible to the fungus than the wild type plants, *cml42* seemed to be more resistant (Fig. 5), indicating that CML37 acts as a positive regulator of the defense against the fungus and CML42 as a negative one. Further effects of *cml37* and *cml42* neutralized each other in the *cml37* × *cml42* double mutant (Fig. 5), providing again evidence that these two CMLs are antagonists in the regulation of this defense reaction.

The signaling pathways leading to defense against herbivores and those that are responsible for the defense against necrotrophs are closely related as both of them rely on the jasmonate pathway (Glazebrook 2005; Howe and Jander 2008; Thomma et al. 1998). Since CML37 and CML42 are influencing this phytohormone pathway [Scholz et al. (2014); Vadassery et al. (2012) and Fig. 3], it is not surprising that they have similar roles in the regulation of the defense against the herbivore *S. littoralis* and the pathogen *A. brassicicola*.

### The antagonism of CML37 and CML42 in the drought stress response

Besides biotic stress, plants have to cope with changing abiotic conditions. The availability of water is one of the major abiotic factors. It is known that CMLs play important roles in regulating the drought stress response. ShCML44, a CML isolated from wild tomato plants (*Solanum habrochaites*), confers to drought tolerance when overexpressed in *Arabidopsis* (Munir et al. 2016)*.* The same effect was shown for the rice (*Oryza sativa*) CML OsMSR2 (Xu et al. 2011). Also CML37 and CML42 have been shown to mediate the drought stress response of *Arabidopsis*, whereby CML37 was revealed as a positive regulator and CML42 as a negative regulator (Scholz et al. 2015; Vadassery et al. 2012). By studying the drought stress response of *cml37* × *cml42*, we could show that CML37 and CML42 are also antagonists in the regulation of the drought stress response. Similar to the response to biotic stress treatments, *cml37* × *cml42* displayed a wild type-like drought stress phenotype (Fig. 6A), suggesting that the opposite effects found in *cml37* and *cml42* neutralize each other in *cml37* × *cml42*.

The response to abiotic stresses is mainly regulated by the phytohormone ABA (Vishwakarma et al. 2017). Both *cml37* and *cml42* displayed altered ABA levels upon drought stress. In *cml42* levels of ABA were increased compared to wild type plants, whereas in *cml37* ABA was not induced at all after drought treatments (Scholz et al. 2015; Vadassery et al. 2012). In line with the drought stress phenotype of *cml37* × *cml42*, plants elevated as much ABA upon drought as wild type plants, confirming the thesis that CML37 and CML42 regulate the drought stress response antagonistically.

### The function of the CML37/CML42 antagonism in plant stress regulation

By investigating a *cml37* × *cml42* double knock out line, we could show that the two Ca^2+^ sensors CML37 and CML42 act antagonistically in both the jasmonate-mediated regulation of biotic as well as ABA-mediated regulation of abiotic stress responses. Whereas CML37 performs in all cases as a positive regulator of the stress responses, CML42 is counteracting as a negative regulator. For the Ca^2+^ binding proteins EHB1 and AGD12, in *Arabidopsis* reduced phototropism and gravitropism in *agd12* mutants but enhanced phototropism and gravitropism in *ehb1* mutants was demonstrated (Michalski et al. 2017; Dümmer et al. 2016).

**Fig. 7:**
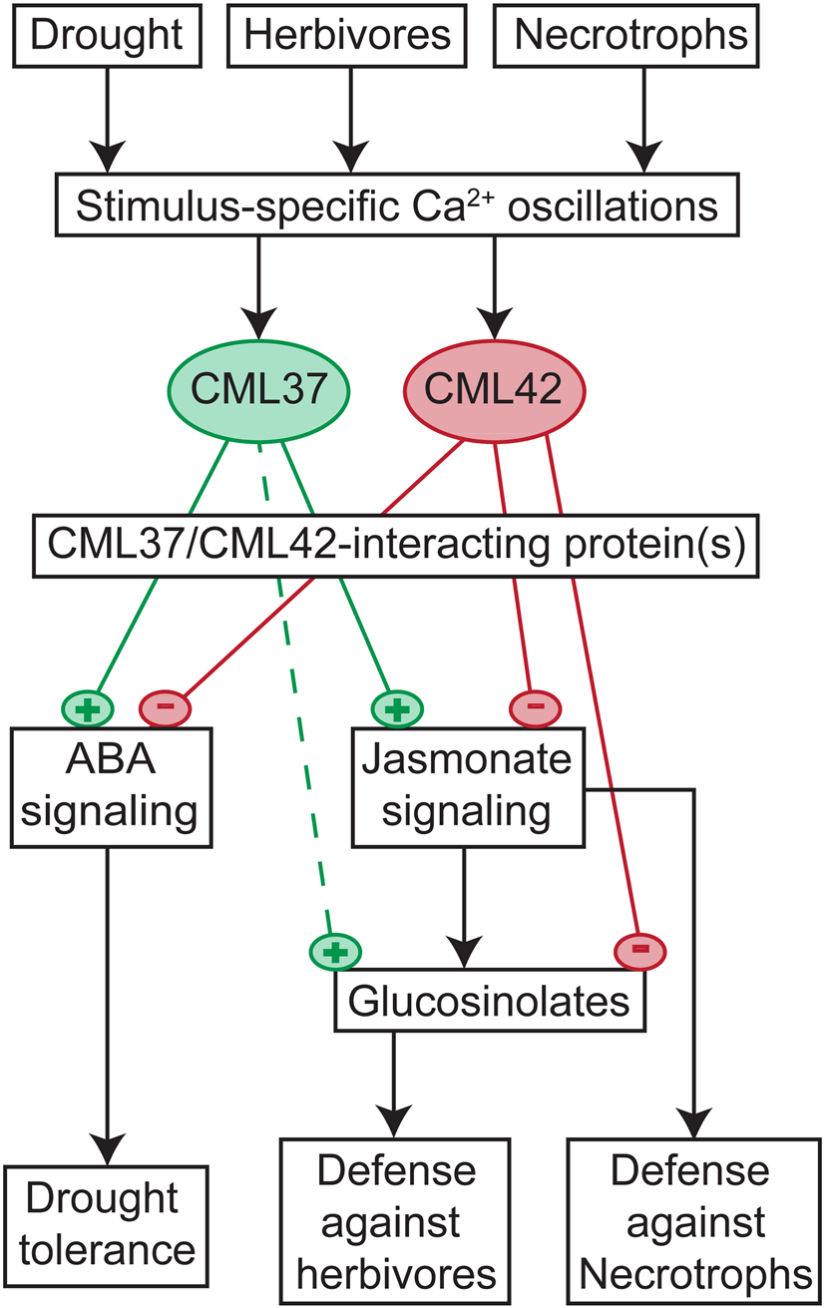
Model for the antagonistic effects of CML37 and CML42 on different stress responses in *Arabidopsis*. CML37 and CML42 are Ca^2+^ sensors that act antagonistically on the plant stress response to drought, herbivores and necrotrophs. After sensing Ca^2+^ elevations in the cell, they bind to yet unknown targets [CML37/CML42 interacting protein(s)] and influence downstream phytohormone pathways. CML37 positively influences the ABA elevation induced by drought stress, enhancing drought tolerance. CML42 negatively affects the drought-induced ABA elevation, and thus the drought tolerance. CML37 has a positive effect on the jasmonate biosynthesis that is counteracted by CML42. CML42 negatively influences jasmonate-dependent responses. Thus CML37 positively influences the defense against herbivores, whereas CML42 has a negative impact. Further CML37 and CML42 play the same roles in the defense against necrotrophic fungi, which might be connected to their effects on the jasmonate signaling pathway. In all shown defense reactions positive and negative effects of CML37 and CML42 are balanced out. CML37 and CML42 might be used for fine tuning these stress responses. Symbols: + means a positive influence,—a negative influence, dashed lines indicate that the influence was proven indirectly by examining the double knock out line of *CML37* and *CML42*

Strikingly, in the *cml37* × *cml42* plants both single effects are balanced out, generating again a wild type-like phenotype (summarized in Fig. 7). Similar effects have been shown for two zinc finger proteins in *Arabidopsis*, LSD1 and LOL1, which antagonistically regulate pathogen-induced cell death: A double knock out of both proteins led to a wild type-like cell death response (Epple et al. 2003). It was hypothesized by the authors that this antagonism is used in the plant to control the cell death response: an imbalance towards the positive regulator would activate the cell death response, whereas an imbalance towards the negative regulator would counteract it (Epple et al. 2003). Similarly, also CML37 and CML42 could fine tune defense responses in the plant. This can be used by the plant also to coordinate competing responses to stresses that occur at the same time. However, further studies dealing with parallel stress treatments are needed to address this hypothesis.

Further, CMLs belong to the class of Ca^2+^ sensor relays and thus just possess the functional domains to bind Ca^2+^ (Sanders et al. 2002). Therefore, to transduce the information given in the Ca^2+^ signature into the appropriate defense response, they need to bind to a certain target protein. For instance, it was shown that the soybean (*Glycine max*) GmCaM1 and GmCML1 (former GmCaM4) antagonistically regulate the activity of the transcription factor MYB2 in *Arabidopsis* (Yoo et al. 2005) and, thus, the salt stress response. Similarly, CML37 and CML42 might influence transcription factors that are known to control the expression of jasmonate or ABA responsive genes. For example, Danisman et al. (2012) showed the antagonistic function of class I and class II TCP transcription factors in the control of leaf development. Furthermore CML37 and CML42 might form heterodimers leading to the antagonistic effects as it known from the above mentioned LSD1 and LOL1 (Epple et al. 2003). To understand the antagonism between the two CMLs in detail, the downstream interacting proteins need to be identified in future projects.

## Data Availability

Data available upon request.
